# Novel integrative models to predict the severity of inflammation and fibrosis in patients with drug-induced liver injury

**DOI:** 10.3389/fmed.2025.1571406

**Published:** 2025-04-28

**Authors:** Yue Zhang, Chuan Lu, Jingying Xu, Qiqi Ma, Mei Han, Li Ying

**Affiliations:** ^1^Department of Gastroenterology, The Second Hospital of Dalian Medical University, Dalian, China; ^2^Department of Cardiology, The Second Hospital of Dalian Medical University, Dalian, China

**Keywords:** drug-induced liver injury, non-invasive evaluation, diagnosis, inflammation, fibrosis

## Abstract

**Background and aims:**

Drug-induced liver injury (DILI) is becoming a worldwide emerging problem. However, few studies focus on the diagnostic performance of non-invasive markers in DILI. This study aims to develop novel integrative models to identify DILI-associated liver inflammation and fibrosis, and compare the predictive values with previously developed indexes.

**Methods:**

A total of 72 DILI patients diagnosed as DILI through liver biopsy were enrolled in this study. Patients were divided into absent-mild (S0–S1, G0–G1) group and moderate–severe (S2–S4, G2–G4) group based on the histological severity of inflammation and fibrosis. We used the area under the receiver operating characteristics curves (AUC) to test the model performances. Backward stepwise regression, best subset and logistic regression models were employed for feature selection and model building. Prediction models were presented with nomogram and evaluated by AUC, Brier score, calibration curves and decision curve analysis (DCA).

**Results:**

For diagnosing moderate–severe inflammation and fibrosis, we calculated the AUC of gamma-glutamyl transpeptidase-to-platelet ratio (GPR), aspartate aminotransferase-to-platelet ratio index (APRI), fibrosis-4 index (FIB-4) and fibrosis-5 index (FIB-5), which were 0.708 and 0.676, 0.778 and 0.667, 0.822 and 0.742, 0.831 and 0.808, respectively. Then, backward stepwise regression, best subset and logistic regression models were conducted for predicting significant liver inflammation and fibrosis. For the prediction of ≥G2 inflammation grade, the AUC was 0.856, 0.822, 0.755, and for the prediction of ≥S2 fibrosis grade, the AUC was 0.889, 0.889, 0.826. Through Brier score, calibration curves and DCA, it was further demonstrated that backward stepwise regression model was highly effective to predict both moderate–severe inflammation and fibrosis for DILI.

**Conclusion:**

The backward stepwise regression model we proposed in this study is more suitable than the existing non-invasive biomarkers and can be conveniently used in the individualized diagnosis of DILI-related liver inflammation and fibrosis.

## 1 Introduction

Drug-induced liver injury (DILI) is a potential and usually unpredictable response that may occur due to the use of many prescribed medicines, herbal products or diet supplements. Previous research on DILI has not been clear enough, and new drugs are constantly being used in clinical practice. Although DILI is generally rare (around 0.3–0.33 ‰ in the healthcare system) (1–3), it is a potential life-threatening adverse drug reaction that accounts for over 50% of acute liver failure cases in Western countries (4). Therefore, DILI has become a global new issue. During long-term follow-up, it was found that a small number of cases may progress to chronic liver injury, which was manifested as asymptomatic elevated liver biochemistry, aggravated chronic hepatitis, and even cryptogenic cirrhosis (5–7). In the advanced stage of DILI, patients have a higher risk of extrahepatic metabolic disorders and complications, so early diagnosis of DILI is of great significance.

The diagnosis of DILI in clinical practice is mainly based on comprehensive analysis of detailed medical history, clinical symptoms, clinical serum biochemical indicators and so on. When patients diagnosed with liver injury suspect a history of drug exposure, non-drug related liver disease should be excluded first. Considering these factors, it is recommended to use the Roussel Uclaf Causal Relationship Assessment Method (RUCAM) scale for scoring and evaluating the possibility of a diagnosis of DILI, but it is still unable to make a definite diagnosis. Liver biopsy (LB) is considered as the “gold standard” for diagnosis, differential diagnosis, and evaluation of liver inflammation and fibrosis. However, this method is an invasive procedure with potential complications and sampling errors (8). More importantly, as an invasive method, it cannot guarantee the dynamic observation of liver fibrosis and inflammation. So far, there is a lack of specific biomarkers for evaluating the severity of inflammation and fibrosis in DILI. While in recent years, researches on non-invasive diagnostic indicators of liver inflammation and fibrosis have attracted attentions. Non-invasive liver fibrosis models composed of simple parameters have been developed to predict liver fibrosis in patients with viral hepatitis, such as fibrosis index based on fibrosis-4 index (FIB-4), aspartate transaminase (AST)-to-platelet ratio index (APRI), gamma-glutamyl transpeptidase-to-platelet ratio (GPR) (9–14). APRI and FIB-4 are recommended methods to determine liver fibrosis and cirrhosis in the World Health Organization (WHO) chronic hepatitis B (CHB) guidelines (15). GPR has been found to be more accurate than APRI and Fib-4 in evaluating liver fibrosis in patients with CHB virus infection (14). Recently, a growing number of studies indicate that a new model, fibrosis-5 index (FIB-5), which combines Alb, platelet count (PLT), alkaline phosphatase (ALP), AST and alanine aminotransferase (ALT) values, is superior to FIB-4 for diagnosing significant hepatic fibrosis in patients with chronic HBV and HCV infections (16, 17). Moreover, previous studies have suggested that GPR and APRI can also be used to predict the severity of liver inflammation in CHB patients (18–20). In most liver diseases caused by various etiologies, liver fibrosis will develop after sustained chronic inflammatory damage to the liver parenchyma. Given that acute DILI lasting for more than 6 months may progress to chronic, which is similar to the disease progression of hepatitis B or C (7), it can be reasonably inferred that these serological markers that can predict liver fibrosis and inflammation in chronic hepatitis B or C infection may also be used for DILI. However, research in this area is very limited up to now. Therefore, in this study, we elucidated the high-risk factors for DILI and developed non-invasive column chart models to predict the histological stages of inflammation and fibrosis. In addition, we also compared the diagnostic accuracy of our new models with GPR, APRI, FIB-4, and FIB-5 to determine the optimal predictive model.

## 2 Methods

### 2.1 Study population

We retrospectively collected the clinical and routine laboratory data of 72 DILI patients who underwent LB at the Second Affiliated Hospital of Dalian Medical University from April 2016 to April 2023. The diagnosis of DILI were based on the RUCAM scale (21). Patients with other underlying chronic liver diseases (e.g., viral hepatitis, autoimmune liver disease, liver cancer, Wilson’s disease) were excluded. The study was carried out in accordance with the Declaration of Helsinki and was approved by the Ethics Committee of the Second Hospital of Dalian Medical university. All patients signed a written informed consent form before LB.

### 2.2 Evaluation of clinical and laboratory data

Patients underwent in-depth medical examinations, including medical history, physical examination, and laboratory test (including blood routine, liver biochemistry and other hematological parameters). All fasting blood samples were taken before LB. The values of GPR, APRI, FIB-4 and FIB-5 were calculated based on the laboratory results (14, 17, 22, 23).

The formulas of model calculations are as follows:


$$GPR=\left(GGT/\mathrm{upper limit of normal for}\;GGT\right)/PLT\;\left(10^{9}/L\right)\times 100$$

$$\mathrm{APRI}=\left[\left(AST/\mathrm{upper limit of normal for}\;AST\right)/PLT\;\left(10^{9}/L\right)\right]\times 100$$

$$\mathrm{FIB}-4=\left(\mathrm{Age}\;\left(\mathrm{year}\right)\times AST\right)/\left(PLT\;\left(10^{9}/L\right)\times \surd \mathrm{ALT}\right)$$

$$\begin{array}{c} \mathrm{FIB}-5=\left(\mathrm{Alb}\;\left(g/L\right)\times 0.3+PLT\;\left(10^{9}/L\right)\times 0.05\right)\\ -\left(ALP\;\left(\mathrm{IU}/L\right)\times 0.014+AST/\mathrm{ALT}\times 6+14\right) \end{array}$$

### 2.3 Assessment of liver histological findings

Ultrasonography-guided percutaneous LB was performed under local anesthesia using a 16-gauge disposable needle. Liver samples (minimum length of 15 mm) containing at least 5 portal tracts were formalin-fixed and paraffin-embedded for histological analysis. In pathological evaluation, the degree of liver inflammation and fibrosis was determined based on the Scheuer scoring standard (24). The assessment of inflammatory activity was evaluated as five different levels: G0 (portal/periportal none or minimal), G1 (portal inflammation, lobular inflammation but no necrosis), G2 (portal/periportal moderate piecemeal necrosis, lobular focal necrosis or acidophil bodies), G3 (portal/periportal moderate piecemeal necrosis, lobular severe focal cell damage), and G4 (portal/periportal severe piecemeal necrosis, lobular damage includes bridging necrosis). The assessment of fibrosis was evaluated as five different levels: S0 (none), S1 (enlarged, fibrotic portal tracts), S2 (periportal or portal-portal septa but intact architecture), S3 (fibrosis with architectural distortion but no obvious cirrhosis), and S4 (probable or definite cirrhosis). According to the histologic severity of liver fibrosis and inflammation, patients were divided into absent-mild (S0–S1, G0–G1) group and moderate–severe (S2–S4, G2–G4) group. The degree of fibrosis in acute DILI was not graded. The pathological diagnosis of each sample was conducted by two pathologists blinded to the patients’ clinical characteristics and laboratory data. In case of discrepancies, a third highly experienced liver pathologist reviewed the pathology specimens and the final results were determined after discussion among all pathologists.

### 2.4 Statistical analysis

Normality test of data was performed by Shapiro–Wilk test. Normal distribution continuous data was presented as mean ± standard deviation and non-normal distribution continuous data was represented by median (25th and 75th percentiles). T-test and the Mann–Whitney non-parametric U-test were used to compare data that conforms to normal distribution and non-normal distribution, respectively. Chi square test was used for classified data. Backward stepwise regression, best subset and logistic regression were performed to investigate the independent variables predictive of significant inflammatory and fibrosis (≥G2 and ≥S2), and nomograms were constructed to display the optimal models. The area under the receiver operating characteristics curve (AUC) was used to assess the diagnostic value of the models. Brier score, calibration curve and decision curve analysis (DCA) were generated for comprehensive evaluation to make more accurate prediction. Statistical analysis was performed with SPSS (version 27) and R software (version 4.2.3). The packages used in this study were “readr,” “Mass,” “rms” and “PROC.” Visualization of receiver operating characteristic (ROC) curve was carried out with the MedCalc Statistical Software version 16.1 (MedCalc Software bvba, Ostend, Belgium). *p* < 0.05 was considered statistically significant.

## 3 Results

### 3.1 Baseline characteristics of patient population

A total number of 72 DILI patients was included in this study. There were 23 cases (31.9%) and 49 cases (68.1%) of patients with absent-mild inflammation (G0–G1) and moderate–severe inflammation (G2–G4), respectively. Among them, 2 cases (2.78%) were classified as G0, 21 cases (29.17%) as G1, 26 cases (36.11%) as G2, 17 cases (23.61%) as G3, and 6 cases (8.33%) as G4. There were 27 patients (54.0%) with absent-mild fibrosis (S0–S1) and 23 patients (46.0%) with moderate–severe fibrosis (S2–S4), respectively. Among them, 12 cases (24%) are classified as S0, 15 cases (30%) as S1, 12 cases (24%) as S2, 11 cases (22%) as S3, and 0 cases (0%) as S4. Compared with absent-mild liver inflammation (G0–G1) group, the red cell distribution width (RDW), ALT, AST, ALP, total bilirubin (TBIL), GPR, APRI and FIB-4 were significantly increased in the moderate–severe inflammation (G2–G4) group. While mean hemoglobin (Hb), PLT, gamma-glutamyl transpeptidase (GGT), albumin (Alb), and FIB-5 in the moderate–severe inflammation (G2–G4) group were significantly decreased. There was no difference in age, gender and globulin level between G0–G1 and G2–G4. A statistically significant increase in age, RDW, GPR, APRI and FIB-4, and a significant decrease in mean Hb, PLT, Alb, and FIB-5 were observed in patients with moderate–severe fibrosis (S2–S4) when compared with absent-mild liver fibrosis (S0–S1). There was no significant differences in the levels of gender, ALT, AST, ALP, GGT, TBIL and globulin between S0–S1 and S2–S4 (Table 1).

**Table 1:** Clinical characteristics of patients with different inflammation and fibrosis groups.

Variables	G0–G1 (*N* = 23)	G2–G4 (*N* = 49)	*p*	S0–S1 (*N* = 27)	S2–S4 (*N* = 23)	*p*
Age (years)	51 (38,61)	59 (48,63)	0.079	45.52 ± 16.83	58.39 ± 8.90	0.006
Male (%)	8 (34.78%)	15 (30.61%)	0.455	10 (37.04%)	9 (30.13%)	0.869
Hb (g/L)	139.00 ± 13.08	130.76 ± 17.02	0.039	138.89 ± 18.33	125.87 ± 13.70	0.005
PLT (×10^9^/L)	227 (199,305)	203 (155,271)	0.020	225.00 (184.00,321.00)	177.00 (135.00,244.00)	0.015
RDW (fL)	13.30 (12.30,14.90)	14.60 (13.10,15.60)	0.017	12.60 (12.30,15.30)	14.90 (14.60,15.80.00)	<0.001
ALT (IU/L)	260.00 (141.00,674.41)	624.74 (316.80,1089.05)	0.009	311.60 (141.00,810.00)	380.00 (253.00,738.00)	0.345
AST (IU/L)	131.00 (53.21,350.25)	623.00 (296.15,867.77)	<0.001	131.00 (58.14,691.00)	474.48 (178.85,659.25)	0.209
ALP (IU/L)	140.50 (113.00,197.63)	235.00 (164.45,304.00)	<0.001	197.63 (140.50,282.73)	199.00 (155.00,352.00)	0.360
GGT (IU/L)	274.41 (110.63,302.58)	231.00 (177.34,375.20)	0.039	178.68 (110.63,291.54)	206.00 (156.52,372.00)	0.189
TBIL (μmol/L)	21.15 (11.64,208.00)	98.00 (28.05,154.06)	0.041	23.26 (12.09,149.30)	71.73 (29.84,141.00)	0.085
Alb (g/L)	40.09 ± 4.91	38.80 ± 5.45	<0.001	42.37 ± 5.93	37.39 ± 5.19	0.003
Globulin (g/L)	28.02 (25.63,30.95)	28.98 (25.97,34.56)	0.328	28.90 (25.70,35.88)	29.27 (26.34,35.00)	0.633
GPR	1.40 (0.67,2.04)	1.84 (1.22,2.94)	0.005	1.09 (0.73,2.38)	1.78 (1.43,2.66)	0.033
APRI	1.23 (0.75,3.01)	7.34 (2.63,11.78)	<0.001	1.23 (0.75,4.94)	5.36 (2.55,10.11)	0.044
FIB-4	1.73 (0.75,2.63)	5.90 (2.78,9.05)	<0.001	1.73 (0.75,3.64)	5.37 (2.54,9.30)	0.003
FIB-5	5.99 (3.84,8.89)	−0.35 (−3.63,3.67)	<0.001	4.74 (2.56,7.96)	−2.11 (−4.64,0.97)	<0.001

### 3.2 Risk factors for significant liver inflammatory and fibrosis for patients with DILI

The factors with statistical differences between two groups were included in the multivariate logistic regression analysis. The predictive factors included in the multivariate logistic regression analysis determining the severity of inflammation and fibrosis are showed in Tables 2, 3. The results indicated that after adjusting for other potent factors, significant liver inflammation (≥G2) was independently correlated with TBIL and Alb (odds ratios of 0.993 and 0.807, respectively, Table 2). In addition, significant fibrosis (≥S2) was independently correlated with PLT and RDW (odds ratios of 0.988 and 1.755, respectively, Table 3). However, logistic regression analysis has limitations, so we tried more predictive models and further compared them to minimize the errors between predicted values and actual data.

**Table 2:** Multivariate regression analysis exploring the predictors determining the severity of inflammation.

	Adjusted		
	OR value	(95% CI)	*p*
Hb	1.001	0.950–1.106	0.963
PLT	0.994	0.985–1.004	0.189
RDW	1.123	0.694–1.817	0.636
ALT	1.000	0.998–1.003	0.686
AST	1.002	0.998–1.005	0.338
ALP	1.009	0.999–1.018	0.078
GGT	0.998	0.993–1.003	0.442
TBIL	0.993	0.986–0.999	0.033
Alb	0.807	0.685–0.951	0.010

**Table 3:** Multivariate regression analysis exploring the predictors determining the severity of fibrosis.

	Adjusted		
	OR value	(95% CI)	*p*
Age	1.058	0.991–1.129	0.089
Hb	0.963	0.908–1.021	0.205
PLT	0.988	0.977–0.998	0.024
RDW	1.755	1.029–2.993	0.039
Alb	0.978	0.809–1.183	0.822

### 3.3 AUCs of GPR, APRI, FIB-4, and FIB-5 were conducted for diagnosing the severity of inflammation and fibrosis in DILI

**Figure 1:**
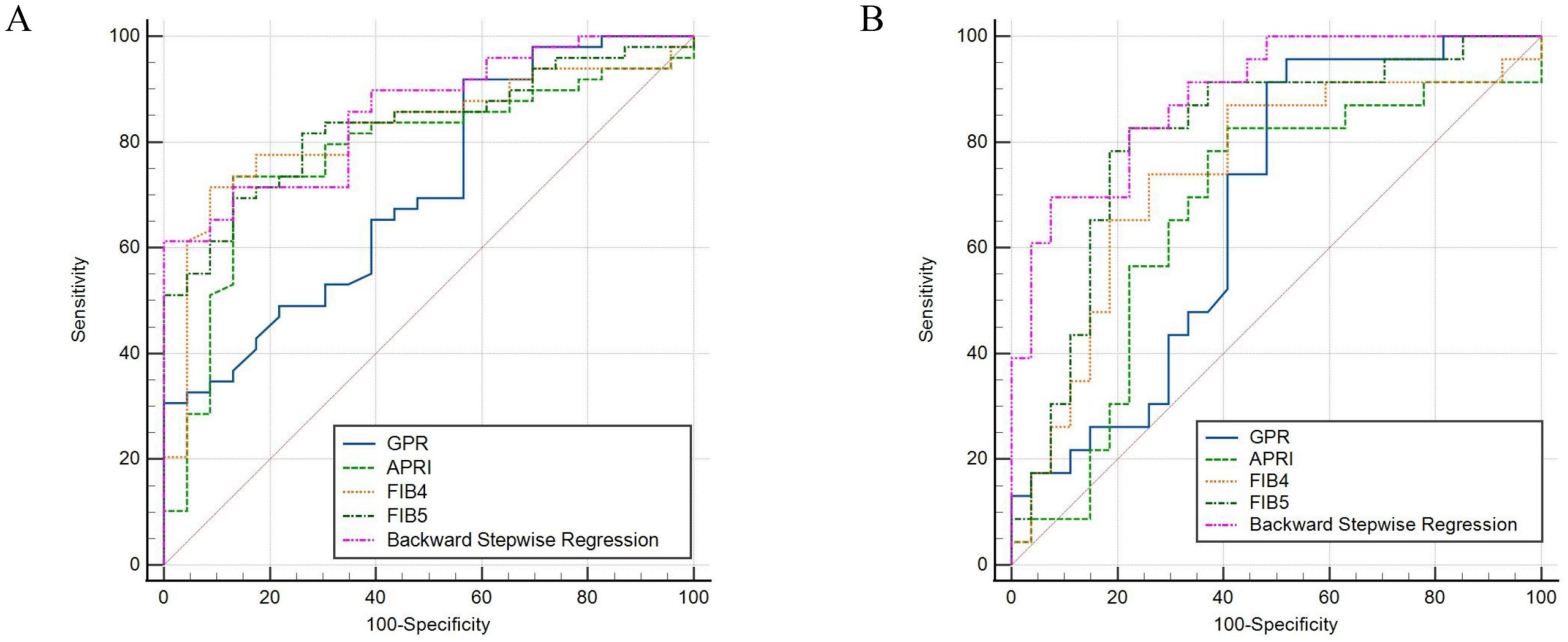
ROC curves of backward stepwise regression model, GPR, APRI, FIB-4, and FIB-5 for diagnosing inflammation ≥ G2 **(A)**, fibrosis ≥ S2 in DILI **(B)**.

AUCs of GPR, APRI, FIB-4, and FIB-5 for predicting moderate–severe inflammation (G2–G4) were 0.708 (95% confidence interval [CI]: 0.589–0.809), 0.778 (95% CI: 0.664–0.867), 0.822 (95% CI: 0.714–0.902), and 0.831 (95% CI: 0.724–0.909), respectively. The AUCs of GPR, APRI, FIB-4, and FIB-5 for predicting moderate–severe fibrosis (S2–S4) were 0.676 (95% CI: 0.528–0.801), 0.667 (95% CI: 0.519–0.794), 0.742 (95% CI: 0.599–0.856), and 0.808 (95% CI: 0.672–0.906), respectively. In order to further explore the diagnostic efficiency of these four models, we compared their AUCs with each other. The results showed that FIB-5 performed better than GPR in predicting G2–G4 inflammation (*p* = 0.041). For predicting S2–S4 fibrosis, FIB-5 performed better than APRI (*p* = 0.009) and GPR (*p* = 0.046), and FIB-4 performed better than APRI (*p* = 0.020). There was no significant difference between the other pairwise comparisons (Table 4; Figures 1A,B). In conclusion, Fib-5 indicated the most excellent clinical value among these four models for predicting both liver inflammation and fibrosis in DILI patients.

**Table 4:** Diagnostic performance of GPR, APRI, FIB-4 and FIB-5 for liver inflammation and fibrosis.

	G2–G4					S2–S4				
	AUC (95% CI)	Sensitivity (%)	Specificity (%)	PPV (%)	NPV (%)	AUC (95% CI)	Sensitivity (%)	Specificity (%)	PPV (%)	NPV (%)
GPR	0.708 (0.589–0.809)	91.84	43.48	77.6	71.4	0.676 (0.528–0.801)	95.65	48.15	61.1	92.9
APRI	0.778 (0.664–0.867)	73.47	86.96	92.3	60.6	0.667 (0.519–0.794)	82.61	59.26	63.3	80.0
FIB-4	0.822 (0.714–0.902)	71.43	91.30	94.6	60.0	0.742 (0.599–0.856) ^b^	73.91	74.07	70.8	76.9
FIB-5	0.831 (0.724–0.909)^a^	69.39	86.96	91.9	57.1	0.808 (0.672–0.906) ^ab^	82.61	77.78	76.0	84.0

^aCompared with AUC of GPR, p < 0.05. bCompared with AUC of APRI, p < 0.05.^

### 3.4 Constructing and validating nomograms for predicting severe liver inflammatory and fibrosis in DILI patients

**Figure 2:**
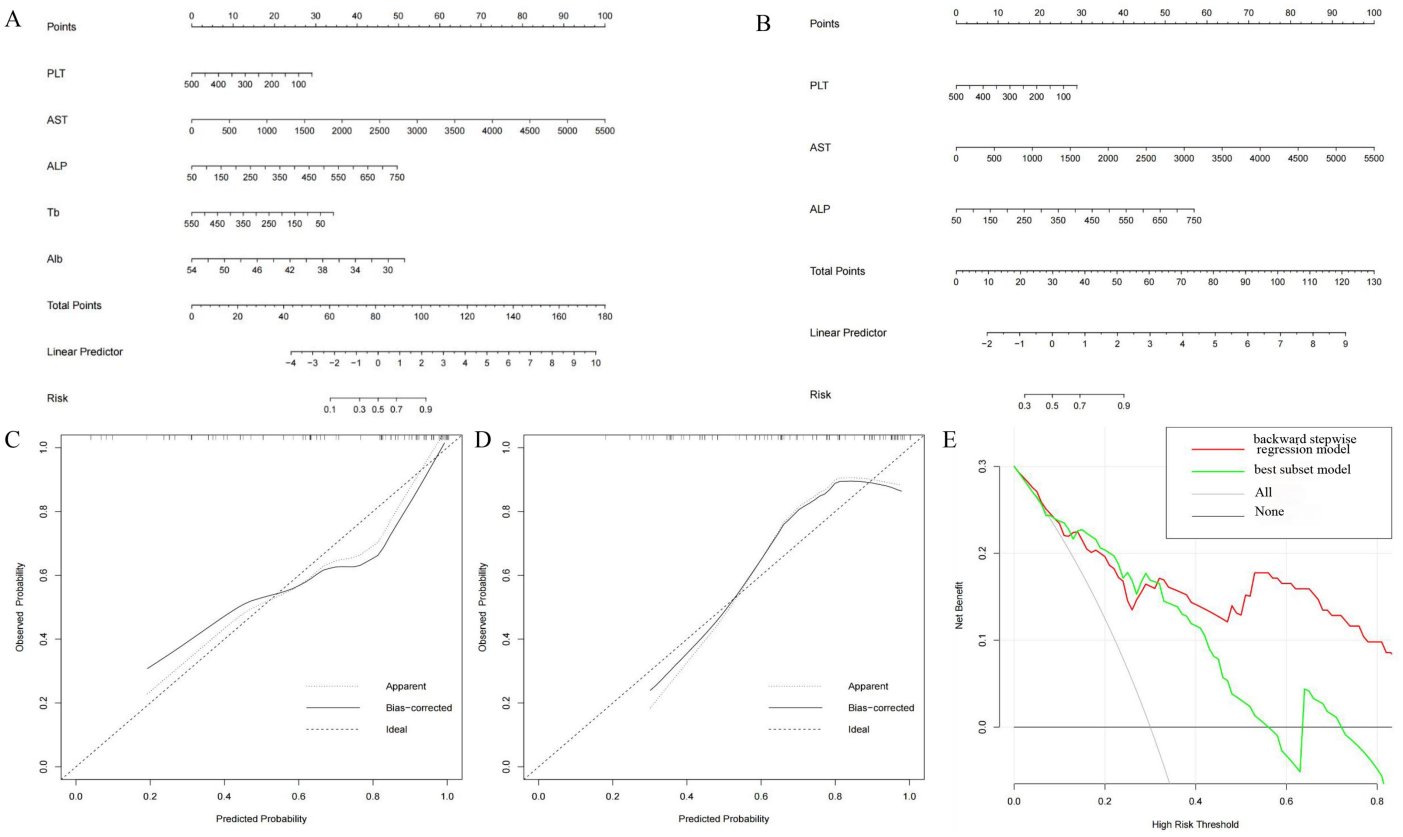
Construction and validation of nomogram for predicting severe liver inflammatory in DILI patients based on backward stepwise regression model and best subset model. Nomogram of backward stepwise regression **(A)** and best subset **(B)**. The calibration curves of nomogram for backward stepwise regression model **(C)** and best subset model **(D)**. DCA of nomogram for backward stepwise regression model and best subset model **(E)**.

**Figure 3:**
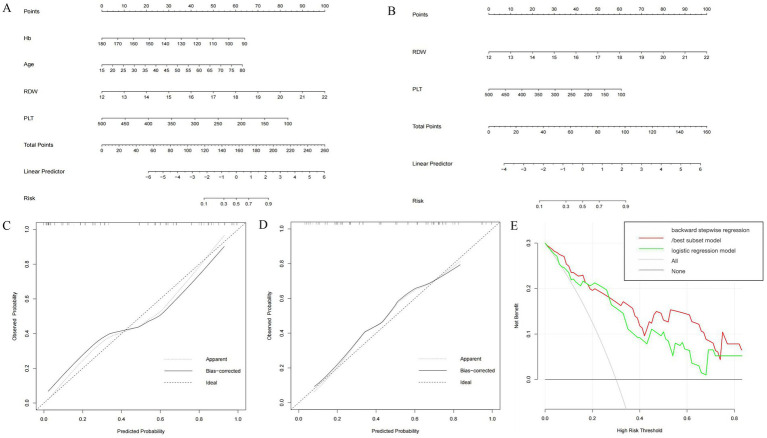
Construction and validation of nomogram for predicting significant liver fibrosis in DILI patients based on backward stepwise regression model, best subset model and logistic regression model. Nomogram of backward stepwise regression /best subset model **(A)** and logistic regression model **(B)**. Calibration curves of nomogram for backward stepwise regression/best subset model **(C)** and logistic regression model **(D)**. DCA of backward stepwise regression model/best subset model and logistic regression model **(E)**.

In order to establish significant predictive models for liver inflammation and fibrosis, we conducted backward stepwise regression, best subset and multivariate logistic regression analysis to select suitable variables. PLT, AST, ALP, Tb and Alb were eventually identified from candidate variables by backward stepwise regression; PLT, AST and ALP were confirmed by best subset; and Tb and Alb were selected by logistic regression, respectively. Then we performed nomograms based on the results of above analysis. In the nomogram, we can sum up the points corresponding to each variable and associate the total points with the risk line at the bottom to assess the probability of severe inflammation and fibrosis (Figures 2A,B, 3A,B). For the study of predicting G2–G4 inflammation, the AUCs of backward stepwise regression model, best subset model, logistic regression model were 0.856 (0.772–0.941), 0.822 (0.706–0.937), 0.755 (0.637–0.873); the Brier scores were 0.141, 0.174, 0.174; the threshold were 0.846, 0.667, 0.815; the specificity were 100, 87.0, 95.7%; the sensitivity were 61.2%, 71.4%, 46.9%, respectively (Table 5). Due to the AUC of logistic regression analysis being 0.755 (0.637–0.873), which was lower than APRI, FIB-4, and FIB-5, this analysis method was not further used to construct the model. We found that AST contributed the most to the backward stepwise regression model, followed by Alb, ALP and TBIL, while PLT accounted for the least ratio (Figure 2A). In the model of best subset, AST contributed the most to the model, followed by ALP and PLT (Figure 2B). In order to clarify the predictive performance of the newly constructed models, we compared their AUCs with those of pre-existing indicators. The AUC of backward stepwise regression model was higher than all these four pre-existing models above (Figure 1A). The calibration curves and DCA of backward stepwise regression model and best subset model were plotted and shown in Figures 2C–E. The calibration curve of backward stepwise regression was more closely aligned with the ideal line than best subset, indicating backward stepwise regression represented better consistency between the predicted and actual results. The DCA showed that the decision curve for backward stepwise regression was further way from the baseline than that of backward stepwise regression, suggesting that the prediction model of backward stepwise regression had more substantial net clinical benefits.

**Table 5:** Diagnostic performance of different models for liver inflammation and fibrosis grades.

	G2–G4					S2–S4				
	AUC (95% CI)	Brier score	Threshold	Specificity (%)	Sensitivity (%)	AUC (95% CI)	Brier score	Threshold	Specificity (%)	Sensitivity (%)
Backward stepwise regression	0.856 (0.772–0.941)	0.141	0.846	100	61.2	0.889 (0.802–0.976)	0.137	0.634	92.6	69.6
Best subset	0.822 (0.706–0.937)	0.174	0.667	87.0	71.4	0.889 (0.802–0.976)	0.137	0.634	92.6	69.6
Logistic regression	0.755 (0.637–0.873)	0.174	0.815	95.7	46.9	0.826 (0.710–0.942)	0.164	0.379	74.1	87.0

For the prediction of S2–S4 fibrosis, Hb, Age, RDW and PLT were eventually identified from candidate variables by both backward stepwise regression and best subset; and RDW and PLT were selected by logistic regression, respectively. The same independent variables were involved in both backward stepwise regression and best subset, so the calculated AUCs of these two models were also the same (0.889 with 95% CI as 0.802–0.976), and the AUC of logistic regression model was 0.826 (95% CI: 0.710–0.942). The Brier score of backward stepwise regression and best subset were the same (both were 0.137), and Brier score of logistic regression model was 0.164. The threshold, specificity and sensitivity of backward stepwise regression (the same as best subset) were 0.634, 92.6 and 69.6%, respectively. The threshold, specificity and sensitivity of logistic regression were 0.379%, 74.1%, and 87.0%, respectively (Table 5). As shown in the Figure 3A, RDW contributed the most to the backward stepwise regression/ best subset model, followed by PLT, Hb, and age accounted for the least ratio. In the model of multivariate logistic regression analysis, RDW contributed the most to the model, followed by PLT (Figure 3B). The AUC of backward stepwise regression model was higher when compared with four pre-existing models mentioned above (Figure 1B). The calibration curves and DCA were plotted and shown in Figures 3C–E. The results indicated that backward stepwise regression model presented better consistency with the ideal line and greater substantial net clinical benefits than best subset model.

## 4 Discussion

DILI is the most common cause of acute liver failure in Western countries (4), so accurately assessing the severity of liver disease in DILI patients is crucial. LB is the gold standard for evaluating liver diseases. However, LB has some drawbacks, such as invasiveness, high cost, potential life-threatening complications, possible sampling errors, and inconsistent abilities and experience among pathologists. Therefore, several models were proposed for predicting liver inflammation and fibrosis. At present, some prediction models constructed using conventional biochemical indicators are mainly used to predict liver fibrosis, and few models are used to predict liver inflammation. Most models seem to perform well in chronic viral hepatitis, but there are rarely applied to DILI patients.

APRI and FIB-4 have been recommended in assessing hepatic fibrosis by CHB consensus guidelines (15, 25). Several studies revealed that the AUCs of APRI for predicting severe fibrosis and cirrhosis in patients with chronic hepatitis C were 0.77 to 0.88 and 0.83 to 0.94, respectively (16, 26). However, in our study on DILI, the AUC of APRI in predicting moderate–severe fibrosis was 0.667, which was lower than previous research. This may be due to the fact that APRI tends to show more accurate results in cirrhosis, but the patients included in our study did not show DILI induced cirrhosis in pathological results. A study of Shoaei et al. (18) on patients with CHB demonstrated that the AUC of APRI for predicting liver inflammation was 0.66, which was lower than ours (0.778). The possible reasons are as follows: First, the criteria of liver pathology score between these two studies was different. In previous studies, Shoaei SD et al. used the Knodell histological activity index to assess the level of necrotic inflammation. While in our study, the Scheuer scoring criteria was applied. Second, mean AST of moderate–severe inflammation group in our study (623 U/L) was higher than that in the study above (97.82 U/L). Due to the limited number of studies reporting the application of APRI in predicting liver inflammation, these results need to be confirmed through large-scale researches.

There have been various conclusions in previous studies regarding the predictive efficacy of different models in diagnosing liver cirrhosis. The AUC of APRI was slightly higher than that of FIB-4 for evaluating cirrhosis, while there was no difference for fibrosis (27). European Association for the Study of the Liver (EASL)-Asociación latin oamericana para el estudio del hígado (ALEH) clinical practice guidelines showed that FIB-4 owned a good accuracy for diagnosing advanced fibrosis. Studies showed that GPR was a more accurate model than APRI and FIB-4 in predicting different fibrosis levels of CHB patients (14, 28). Shiha et al. (17) studied 604 patients with CHB and found that FIB-5 was superior to FIB-4 in distinguishing between significant and non-significant fibrosis. Similarity, Metwally et al. (16) concluded that FIB-5 was more specific than FIB-4 in evaluating hepatic fibrosis in CHB patients. In our study, we reached similar conclusions. We found that the AUC value of FIB-5 was higher than that of GPR, APRI and FIB-4 in the diagnosing moderate–severe inflammation (G2–G4) and fibrosis (S2–S4) for DILI.

In this study, backward stepwise regression model, best subset model and logistic regression model for predicting significant liver inflammation and fibrosis were created with the available laboratory parameters from DILI. Nomograms were also built by the selected independent variables to predict the risk of moderate–severe inflammation and fibrosis in DILI. The four factors including RDW, PLT, Hb and age built the backward stepwise regression model to predict severe fibrosis. Previous studies indicated that RDW levels were positively relevant to the liver disease severity with various etiologies (29–31), and lower Hb, higher bilirubin and thrombocytopenia are independent predictors of poor outcomes (32, 33), which is consistent with our findings. To compare the clinical usability of these models, Brier score, calibration curve and DCA were performed. By testing the above methods, we found that the model constructed by backward stepwise regression is very effective in predicting moderate–severe inflammation and fibrosis in DILI. Compared with other traditional criteria, such as GPR, APRI, FIB-4 and FIB-5, backward stepwise regression model owns extraordinary predictive power with the AUC more than 0.85. To our knowledge, it is the first time to perform models constructed by various methods and compared them with the existing models to predict significant liver inflammation and fibrosis for DILI. Considering the accuracy range of LB, an AUC > 0.90 cannot be achieved even for a perfect biomarker (34). EASL-ALEH clinical practice guidelines has reported that the AUC below 0.80 is generally considered too imprecise and of no value in clinical practice (35). Our nomogram models are performed by combining several clinical characteristics and have been proved to be highly effective in predicting severe liver inflammation and fibrosis with AUC values of 0.856 and 0.889, respectively. In addition, our nomogram models also have some advantages when compared with other traditional criteria. First of all, nomogram is a user-friendly method that guides clinical decision-making for doctors, as they can transform statistical prediction models into single numerical estimates of event probabilities. By summing the scores of different predictive variables, clinicians can quickly estimate an individual risk of patients without complex calculations. Nomogram integrates multiple clinical variables (e.g., general information, laboratory results), providing a more precise prediction compared to single-factor assessments. Secondly, our models are more accurate to predict severity of liver pathology of patients compared to traditional models.

There are also several limitations in our study. Firstly, potential selection bias is inevitable as our analysis is a retrospective analysis with a single center study. Therefore, the conclusions drawn from this study need to be validated through large-scale prospective studies. Secondly, we did not investigate the previous medication use, which may affect the AST values included in this study. Therefore, similar potential confounding factors should be minimized as much as possible in future large-scale analysis. Thirdly, due to the relatively small sample size, the diagnostic efficiency of the model cannot be verified through the test set, so the predicting value of models need to be validated through large-scale research in the future.

However, our analysis has several highlights. Most studies on non-invasive liver models have been mainly focused on patients with chronic HBV and HCV infection, while little attention was focused on exploring predictive factors for inflammation and fibrosis grades in DILI patients. To our knowledge, this is the first study conducted in DILI patients aimed at determining the clinical usefulness of non-invasive serum markers based models of GPR, APRI, Fib-4, and Fib-5 in inflammation and fibrosis severity, and comparing them with liver biopsy results. Furthermore, the models constructed in our study were compared with GPR, APRI, FIB-4, and FIB-5 models to analyze the diagnostic effectiveness of predicting the severity of inflammation and fibrosis in DILI patients. In conclusion, our models will improve diagnostic accuracy and clinical management, and our findings will help clinicians identify DILI patients with potential risk of severe liver inflammation and fibrosis.

## 5 Conclusion

In summary, our study proposed clinical predicting models for evaluating liver inflammation and fibrosis in DILI. The results indicate that backward stepwise regression model possesses a better clinical application value. Our research can help assess the severity of liver inflammation and fibrosis in clinical practice, while more cohort studies are still needed to validate the accuracy of our predictive model.

## Data Availability

The raw data supporting the conclusions of this article will be made available by the authors, without undue reservation.

## Glossary

Alb: Albumin
ALEH: Asociación latin oamericana para el estudio del hígado
ALP: Alkaline phosphatase
ALT: Alanine aminotransferase
APASL: Asian-Pacific Association for the Study of the Liver
APRI: Aspartate aminotransferase-to-platelet ratio index
AST: Aspartate aminotransferase
AUC: The area under the receiver operating characteristics curve
CHB: Chronic hepatitis B
CI: Confidence interval
DILI: Drug-induced liver injury
DCA: Decision curve analysis
EASL: European Association for the Study of the Liver
FIB-4: Fibrosis-4 index
FIB-5: Fibrosis-5 index
GGT: Gamma-glutamyl transpeptidase
GPR: Gamma-glutamyl transpeptidase-to-platelet ratio
Hb: Hemoglobin
HBV: Hepatitis B virus
HCV: Hepatitis C virus
LB: Liver biopsy
PLT: Platelet count
RDW: Red cell distribution width
ROC: Receiver operating characteristic
RUCAM: Roussel Uclaf Causality Assessment Method
TBIL: Total bilirubin
WHO: World Health Organization
